# miRNA, lncRNA and circRNA: Targeted Molecules Full of Therapeutic Prospects in the Development of Diabetic Retinopathy

**DOI:** 10.3389/fendo.2021.771552

**Published:** 2021-11-10

**Authors:** Xingyu Chang, Guomao Zhu, Zongyan Cai, Yaqi Wang, Rongna Lian, Xulei Tang, Chengxu Ma, Songbo Fu

**Affiliations:** ^1^ The First Clinical Medical College, Lanzhou University, Lanzhou, China; ^2^ The Second Clinical Medical College, Lanzhou University, Lanzhou, China; ^3^ Gansu Province Clinical Research Center for Endocrine Disease, Lanzhou, China; ^4^ Endocrine Disease Clinical Medical Research Center of Gansu Province, Lanzhou, China

**Keywords:** diabetic retinopathy, miRNA, lncRNA, circRNA, diabetes

## Abstract

Diabetic retinopathy (DR) is a common diabetic complication and the main cause of blindness worldwide, which seriously affects the quality of life of patients. Studies have shown that noncoding RNA (ncRNA) has distinct differentiated expression in DR and plays an important role in the occurrence and development of DR. ncRNAs represented by microRNAs (miRNAs), lncRNAs (lncRNAs), and circRNAs (circRNAs) have been shown to be widely involved in the regulation of gene expression and affect multiple biological processes of retinopathy. This article will review three RNAs related to the occurrence and development of DR on the basis of previous studies (especially their effects on retinal microangiopathy, retinal pigment epithelial cells, and retinal nerve cells) and discuss their underlying mechanisms and connections. Overall, this review will help us better understand the role of ncRNAs in the occurrence and development of DR and provide ideas for exploring potential therapeutic directions and targets.

## Introduction

**Figure 1:**
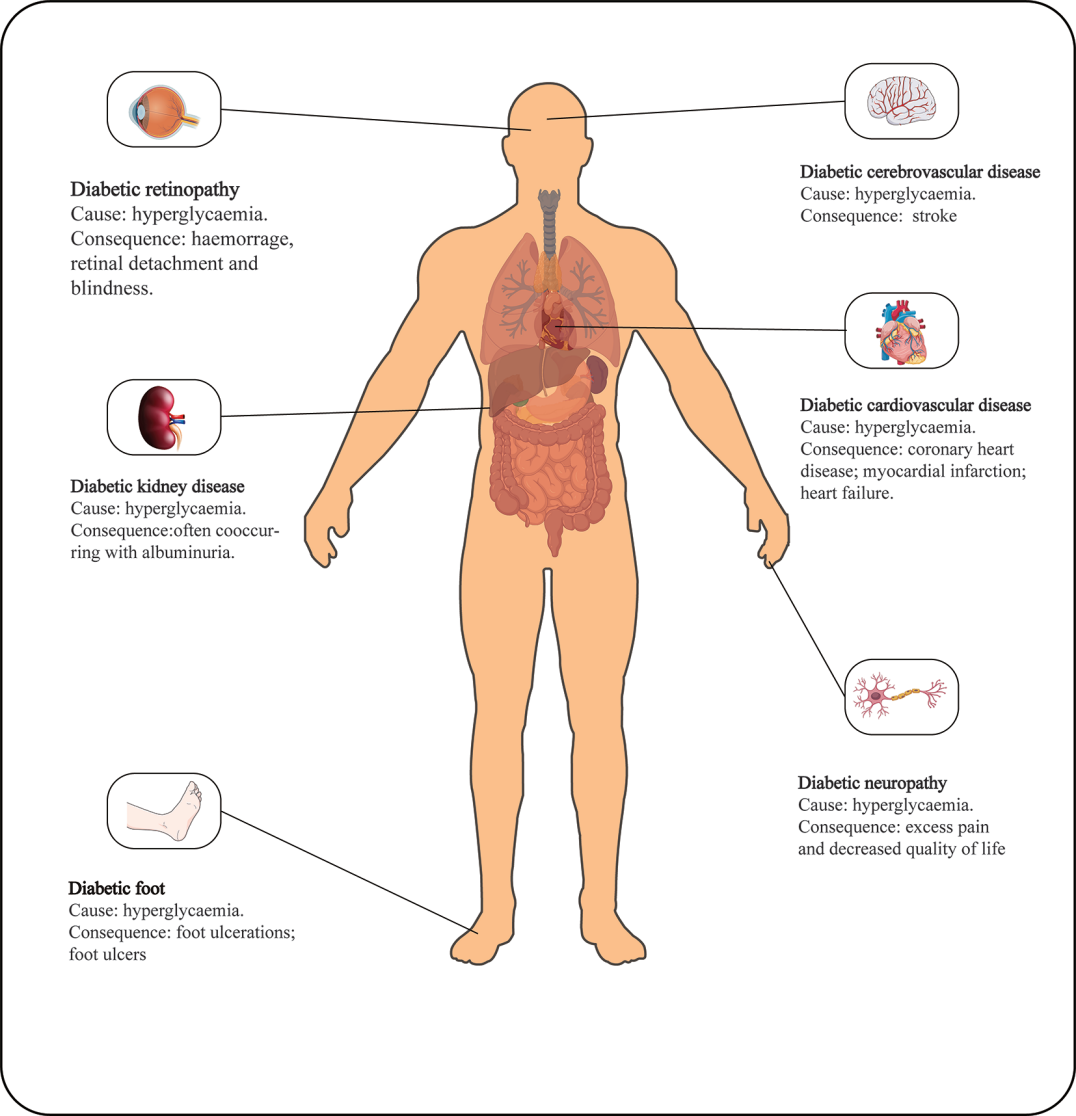
Main causes and consequences of DM.

Diabetic retinopathy is one of the most common microvascular complications of type 1 and type 2 diabetes (**Figure 1**) and is the leading cause of blindness worldwide (1, 2). As the social environment changes, the impact of DR on human vision has become increasingly prominent. There is evidence that the current prevalence of DR in the United States is approximately 35% (3, 4). In the past 30 years, the prevalence of diabetic retinopathy has increased by 7.7%, but the crude prevalence of other blinding diseases has decreased (5). As a complication of a chronic disease, the prevalence and severity of DR are positively correlated with age and the course of the disease (6). The relatively long course of DR can be divided into mild DR or nonproliferative DR (NPDR), characterized by an increase in the number of microaneurysms, and proliferative DR (PDR), characterized by the formation of new blood vessels on the posterior surface of the retina and vitreous, which is more severe than NPDR. Increased vascular permeability, macular edema, retinal distortion and detachment caused by angiogenesis and fibrous tissue contraction and neovascular bleeding are all progressive pathological changes in the development of DR under continuous hyperglycemia stimulation (7). Without treatment, most patients with DR may lose vision within 5-10 years of diagnosis. Currently, there is no optimal clinical treatment method. Vascular endothelial growth factor (VEGF) is considered a promising target, and research has found that VEGF may influence inflammation in DR (8). VEGF can only achieve good curative effects in the late stage of DR, however, and VEGF administration is cumbersome, which does not lead to an ideal treatment method. Therefore, there are broad prospects for the discovery of new therapeutic target molecules and research over therapeutic applications.

A number of studies have confirmed that the pathological process of diabetic retinopathy is related to the differential expression of vascular dysfunction-related proteins, such as VEGF, transforming growth factor (TGF), and sirtuin (SIRT), and their expression levels are closely related to oxidative stress, apoptosis and angiogenesis in the course of DR (9–12). miRNAs, lncRNAs, and circRNAs can be used as upstream regulators or interacting elements to participate in their functioning process. Previous studies have found that 131 circRNAs (13), 8miRNAs (14), and 427 lncRNAs with expression differences during the development of DR compared with normal tissues (15), which suggests that the three RNAs molecules are closely related to the occurrence and development of DR. This review reveals the influence of miRNAs, circRNAs, and lncRNAs on the development of DR, which may increase our understanding of the role of these important molecules and provide new perspectives for clinical DR molecular targeted therapy.

### miRNA

miRNAs are single-stranded ncRNAs consisting of 20-24 nucleotides. In the classical pathway, miRNA is a primary miRNA transcript formed by RNA polymerase II transcription of precursor miRNA genes. After the stem-loop structure is formed, it is cleaved into precursor miRNA and proceeds to mature miRNA by the Disher enzyme (16–18). miRNA can interact with the 3’-untranslated region (UTR) of targeted mRNA through its 6 nt seed sequence, mediate posttranscriptional gene silencing (PTGS) in the cytoplasm (19), and participate in almost all cellular activity regulation, including cell proliferation, migration, differentiation and apoptosis (20, 21). An increasing number of studies have demonstrated that miRNAs play an indispensable role in the development of DR lesions.

### CircRNA

CircRNA was first discovered by Hsu in 1979 to be expressed in the cytoplasm of mammals (22). CircRNAs are a new type of ncRNA generated from linear precursor mRNAs *via* nonclassical splicing (23). Because its closed loop structure is difficult to degrade by nucleases, circRNA is more stable than its linear transcript (24). The composition of circRNA can be divided into exon circRNA (ecircRNAs), intron circRNA (ciRNAs), and exon and intron circRNA (elciRNAs) (25). It has been confirmed that circRNAs mainly regulate the physiological and pathological functions of cells in four ways: serving as miRNA sponges, protein regulators, translation templates and gene expression regulators (23).

### LncRNA

LncRNAs are noncoding protein transcripts composed of more than 200 nucleotides. Studies have shown that a small number of lncRNAs have the potential to encode proteins (26), but this is not the main role of lncRNA in the regulation of cell life activities. LncRNAs mainly regulate gene expression at the pretranscription, posttranscription and posttranscriptional levels, such as epigenetic regulation (group protein methylation, and chromatin remodeling), regulatory transcription factors, endogenous competitive RNA, and antisense lncRNAs (27–29). In many diseases, lncRNAs are differentially expressed, and DR is no exception. Gene therapy based on lncRNAs is an emerging disease treatment strategy and has great potential in the treatment of DR.

## Three Types of RNA Influence the Development of DR Through Microvascular Endothelial Cells

**Figure 2:**
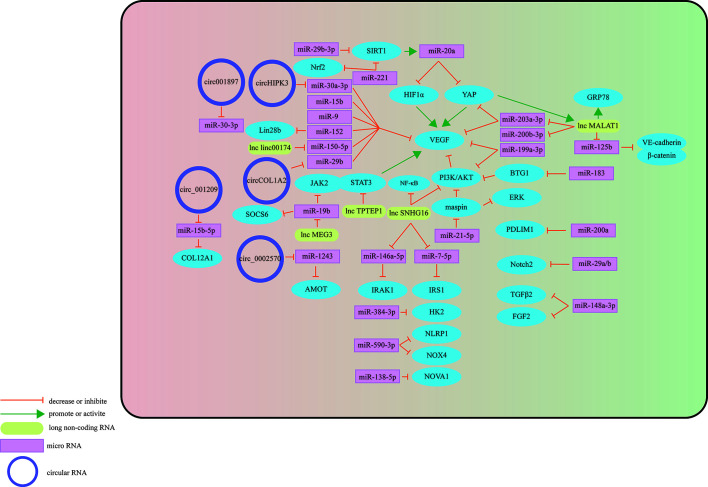
Schematic overview of miRNAs, lncRNAs, and circRNAs affecting vascular endothelial cells and contributing to the pathogenesis of DR.

The retinal vessel wall is composed of three layers: intima, media, and adventitia. The innermost intima consists of a single layer of endothelial cells in direct contact with the vascular cavity, and the media is composed of multiple layers of smooth muscle cells and pericytes. The blood basement membrane forms an outer membrane on the outside (30, 31). Retinal vascular endothelial cells (HRVECs) in the HG state will activate, proliferate and migrate abnormally, which may lead to changes in retinal vascular function (32). Increasing evidence shows that three types of RNAs have important regulatory effects on the process of HG-stimulating dysfunction of retinal vascular endothelial cells (**Figure 2**).

### miRNA-Mediated Mechanism

Some miRNAs may target specific molecules to protect retinal microvascular endothelial cells (RMECs) in DR. The most important target is VEGF. For example, miR-29b-3p can negatively regulate the expression levels of VEGFA and platelet-derived growth Factor B (PDGFB), inhibiting cell cycle progression by decreasing the expression levels of cell cycle-related proteins (cyclins A2, D1, and E1), which prevents the abnormal proliferation of RMECs (33). Previous studies have also found that miR-29b-3p can downregulate SIRT1 to promote RMEC apoptosis (34). However, Pan et al. found that SIRT1 can positively regulate the expression of miR-20a so that miR-20a induces YAP/HIF1α to downregulate VEGF activity and hinder the abnormal proliferation of RMECs in the HG state, suggesting that the restoration of SIRTI may protect RMECs in the HG state (35). MiR-199a-3p might be another target for regulating VEGF. It can downregulate the expression of VEGF and inhibit the PI3K/AKT pathway related to VEGF, protecting RMECs from HG (36). miR-203a-3p is downregulated under the induction of HG, and high expression can decrease the expression levels of VEGFA and HIF-1α to reduce the pathological retinal angiogenesis of PDR (37). Previous studies have shown that miR-15b (38), miR-9 (39), and miR-152 (40) can directly or indirectly downregulate VEGF in RMECs treated with HG, reduce the proliferation of endothelial cells, and hinder the progression of PDR. In addition to VEGF, many miRNAs protect RMECs in the DR state through other signaling pathways. PDLIM1 is a cytoskeletal protein associated with stress fibers, and miR-200a can downregulate the expression of PDLIM1 in RMECs and reduce the apoptotic state of RMECs, decreasing retinal microvascular leakage (41), which implies that miR-200a may be an efficient therapeutic target for the early stage of DR. miR-148a-3p specifically binds to the 3’ untranslated regions of TGFB2 and FGF2, downregulates their expression, and reduces the apoptosis of microvascular epithelial cells in the HG state (42). OPN regulates the inflammatory response at multiple levels (43). In the capillary endothelial cells of DR, it can inhibit miR-29a, indirectly increase the synthesis of type IV collagen, thicken the basement membrane, and aggravate the pathological changes of DR (44). Research in recent years found that miR-29a/b (45), miR-590-3p (46), miR-138-5p (47), and miR-384-3p (48) all have the ability to protect microvascular endothelial cells in DR.

In addition to the protective effect, some miRNAs regulate downstream signals to make RMECs proliferate, promote the formation of new blood vessels, and aggravate the PDR process. HG upregulates miR-183, reduces the expression of BTG1, and activates VEGF-related pathways to upregulate VEGF expression and promote the proliferation of diabetic retinal vascular endothelial cells (49). miR-21-5p is the upstream signal of maspin. After high expression, it enhances the cellular viability of RMECs and promotes the angiogenesis of PDR (50). Some miRNAs promote RMEC apoptosis and aggravate the progression of PDR in the HG state. Overexpressed miR-221 can bind to the 3’UTR of SIRT1 mRNA and downregulate its expression, blocking the Nrf2 pathway and increasing the apoptosis of RMECs (51).

In summary, miRNAs have broad potential in DR treatment, but the therapeutic effect may be difficult to grasp. For example, miR-148a-3p, which is downregulated in RMECs under DR conditions, can hinder the formation of new blood vessels after upregulation, but it will also damage the blood-retinal barrier (42).

### CircRNA-Mediated Mechanism

The differential expression of circRNAs in HRVECs has a significant impact on the progression of DR, which may cause vascular dysfunction and/or promote the formation of new blood vessels and participate in nonproliferative and proliferative DR lesions. Overexpressed circ_001209 serves as a miR-15b-5p sponge in HRVECs, indirectly regulating the expression of COL12A1, causing vascular endothelial cell dysfunction, significantly thinner retinal thickness, and increased apoptosis (52). Similarly, the expression of circHIPK3 located in the cytoplasm of HRVECs increased, downregulating the activity of miR-30a-3p and thereby upregulating the expression of VEGFC, FZD4 and WNT2. Knockout of circHIPK3 can reduce the abnormal proliferation, migration and tubular formation of HRVECs cultured *in vitro*, decreasing retinal acellular capillaries and vascular leakage (53). Therefore, circHIPK3 may play a role in the progression of NPDR. As a sponge of miR-29b, circCOL1A2 promotes the proliferation of retinal microvascular endothelial cells (HRMECs) and inhibits cell apoptosis under the action of VEGF (54). Tissue-specific expression of angiomotin can promote the proliferation of vascular endothelial cells and make them tubular. hsa_circ_0002570 is an inhibitor of miR-1243, which upregulates the expression of angiomotin and promotes the formation of new blood vessels in DR (55). circ001897 is circRNA whose expression level in DR tissues is significantly higher than that in the control group. By targeting miR-30-3p and downregulating its expression level, it can achieve the effect of HRMEC migration and proliferation (56). However, the downstream targeting molecule of miR-30-3p has not yet been determined.

In addition to vascular endothelial cells, circRNAs also play an important regulatory role in pericytes. The histopathological feature in the earliest stage of DR is the loss of pericytes (57). Under the stimulation of diabetes, CZNF532 acts as a sponge of miR-29a-3p to induce increased expression of NG2, LOXL2 and CDK2 to protect retinal pericytes and alleviate pericyte degeneration and vascular dysfunction (58). Therefore, cZNF532 is essential for maintaining pericyte function and vascular homeostasis. Similarly, cPWWP2A in the cytoplasm of pericytes acts as an endogenous miR-579 sponge to inhibit miR-579 activity, which increases the expression of angiopoietin 1, occludin, and SIRT1 (59). Intervening in the expression of cPWWP2A or miR579 may be a treatment strategy for diabetic microvascular complications. A major cause of vascular dysfunction is abnormalities in pericyte-endothelial cell crosstalk (60). There is evidence that circRNAs may be involved in this pathway. CircEhmt1, which is transferred from pericytes to endothelial cells through exosomes, is highly expressed in the nucleus of pericytes and upregulates the level of transcription factor (NFIA), which ultimately inhibits the production of the NLRP3 inflammasome. Activation of the HIF pathway may play an important role in the activation of the NFIA/NLRP3 pathway.

In summary, circRNAs can regulate the functional changes of endothelial cells and pericytes in the DR state through different pathways (**Table 1**), possibly due to the different roles that endothelial cells and pericytes play in neovascularization. DR damage is the main pathway by which circRNAs regulate endothelial cells, while DR protection is the main counterpart in pericytes. The development of circRNA-targeted drugs is helpful for the derivation of new strategies for the clinical treatment of DR.

**Table 1:** The role of miRNA, lncRNA and circRNA in DR and their mechanisms through vascular endothelial cell and targets.

ncRNA	Name	Dysregulation	Possible signaling pathways	Pathogenic functions	Reference
circRNA	circ_001209	upregulated	miR-15b-5p/COL12A1	promote invasion, migration and angiogenesis of HRVECs	(52)
	circHIPK3	upregulated	MiR-30a-3p	promote cell viability, proliferation, migration, and tube formation of HRMECs	(53)
	circCOL1A2	upregulated	miR-29b/VEGF	promote proliferation, migration, angiogenesis and vascular permeability of HRMECs	(54)
	hsa_circ_0002570	upregulated	miR-1243/angiomotin	promote invasion, migration and angiogenesis of HRMECs	(55)
	circ001897	upregulated	miR-30-3p	promote proliferation and migration of HRVECs	(56)
lncRNA	MALAT1	upregulated	miR-203a-3p, GRP78, miR‐200b‐3p/VEGFA	promote migration and angiogenesis of HRMECs	(61–64)
	linc00174	upregulated	miR-150-5p/VEGFA	promote proliferation, migration and angiogenesis of HRMECs	(65)
	SNHG16	upregulated	miR-146a-5p/IRAK1, miR-7-5p/IRS1, NF-kB, PI3K/AKT	promote the angiogenesis of HRMECs	(66, 67)
	TPTEP1	downregulated	STAT3/VEGFA	inhibit viability, migration, and angiogenesis of HRVECs	(68)
	MEG3	downregulated	miR-19b/SOCS6, JAK2/STAT3	inhibit apoptosis and inflammation of HRMECs	(69)
miRNA	miR-29b-3p	upregulated	SIRT1, PDGFB, VEGFA	promote apoptosis of HRMECs	(33, 34)
	miR-20a	downregulated	YAP/HIF1α/VEGFA	promote proliferation and angiogenesis of RMECs	(35)
	miR-199a-3p	downregulated	VEGF/PI3K/AKT	inhibit migration and angiogenesis of HRMECs	(36)
	miR-203a‐3p	downregulated	VEGFA and HIF‐1α	inhibit proliferation, migration and angiogenesis of HRMECs	(37)
	miR-15b	downregulated	VEGFA	inhibited proliferation of HRMECs	(38)
	miR-9	downregulated	VEGFA	inhibit proliferation and angiogenesis of RMECs	(39)
	miR-152	downregulated	Lin28b/VEGF	inhibit angiogenesis of HRMECs	(40)
	miR-183	upregulated	BTG1, PI3K/Akt/VEGF	inhibit proliferation and angiogenesis of RMECs	(49)
	miR-200a	downregulated	PDLIM1	inhibit viability, apoptosis and migration of HRMECs	(41)
	miR-148a-3p	downregulated	TGFβ2 and FGF2	inhibit apoptosis and angiogenesis of HRMECs	(42)
	miR-29a	upregulated	Col IV	promote proliferation and angiogenesis of HRMECs	(44)
	miR-29a/b	downregulated	Notch2	inhibit endothelial-mesenchymal transition of HRMECs	(45)
	miR-590-3p	downregulated	NLR,NOX4/ROS/TXNIP/NLRP3	inhibit pyroptosis of HRMECs	(46)
	miR-138-5p	downregulated	NOVA1	inhibit proliferation of RMECs	(47)
	miR-384‐3p	downregulated	HK2	inhibit proliferation and tube formation of RMECs	(48)
	miR-21-5p	upregulated	maspin, PI3K/AKT, ERK	promote proliferation and angiogenesis of HRMECs	(50)
	miR-221	upregulated	SIRT1/Nrf2	promote apoptosis of HRMECs	(51)

### LncRNA-Mediated Mechanism

Existing studies have shown that adjusting the expression of specific target mRNAs or miRNAs enables lncRNAs to regulate endothelial cell diseases. In 2014, studies found that lncRNA MALAT1 may cause damage to diabetic endothelial cells (70). Recently, the damage mechanism of MALAT1 in DR vascular endothelial cells was further explored, and it was discovered that it may promote the development of DR by mediating the activation and inhibition of different pathways. Highly expressed MALAT1 is involved in HG-induced angiogenesis, ERS and inflammation, which may be achieved by MALAT1’s inhibition of the expression of GRP78 (61). Other studies have found that highly expressed YAP1 may upregulate the expression of MALAT1 to promote the proliferation of endothelial cells, which may be the effect of downregulating miR-200b-3p and increasing the expression of VEGF simultaneously (62). miR-125b has also been proven to be a downstream targeting molecule of MALAT1. The decreased expression of miR-125b can upregulate the expression of the VE-cadherin/MALAT1-catenin complex and neovascularization-related proteins to participate in the process of DR (63). miR-203a-3p is also a downstream target molecule of MALAT1 that has been explored in HRMECs under HG treatment (64), but the downstream signal of miRNA needs to be further studied. There are many other lncRNAs involved in the pathological process of HRMEC. Upregulated Linc00174 can target miR-150-5p, upregulate VEGF, and promote the angiogenesis of DR (65). Similarly, SNHG16 is also upregulated in HG-induced vascular endothelial cells and activates the NF-kB pathway through miR-146a-5p/IRAK1 and miR-7-5p/IRS1, with the NF-kB pathway triggered by the PI3K/AKT pathway, promoting hRMEC dysfunction (66). In addition to promoting HRMEC lesions induced by HG, some lncRNAs have a protective effect on HRMECs. As an upstream regulator of miR-195, after upregulating SNHG16, H2O2-induced tube formation of HMRECs was significantly inhibited (67). Likewise, TPTEP1 can inhibit the phosphorylation and nuclear translocation of STAT3, thereby downregulating VEGFA mRNA and protein levels and hindering the formation of new blood vessels under HG conditions (68). MEG3 negatively regulates miR-19b to inhibit HG-induced inflammatory factors generated from HMRECs, caspase-3/7 and cell apoptosis (69). Therefore, reversing the expression level of lncRNAs under certain conditions may play a role in promoting the alleviation of DR.

## Three Types of RNA Influence the Development of DR Through Retinal Pigment Epithelial Cells

**Figure 3:**
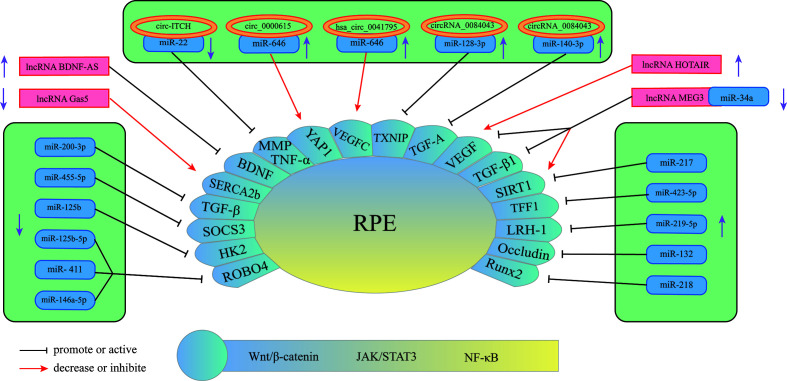
Schematic overview of miRNAs, lncRNAs, and circRNAs affecting RPE and contributing to the pathogenesis of DR.

The retinal pigment epithelium (RPE) is a lifelong layer of highly polarized pigment epithelial cells located between the photoreceptors and the choroid (71). The RPE participates in the formation of the external visual blood barrier, and the melanin particles in RPE can absorb light to protect it from lesions due to photooxidative stress (72). In addition to phagocytosis, degradation of the photoreceptor outer ganglion (POS) terminal, maintenance of the retinoic acid cycle, and protection against light and oxidative stress, RPE can also secrete proteins in a polarized manner; for example, vascular endothelial growth factor (VEGF) is secreted mainly toward the basal direction, which is essential for the growth of choroidal blood vessels (73) and is also an important factor in promoting the progression of DR in the proliferative phase. An increasing number of studies have found that miRNAs, lncRNAs, and circRNAs are differentially expressed in RPE induced by HG and have certain effects on its pathological changes (**Figure 3**) (**Table 2**). Therefore, understanding the role of these three RNAs in RPE lesions may help us to understand the development of DR.

**Table 2:** The role of miRNA, lncRNA and circRNA in DR and their mechanisms through retinal pigment epithelial cell and targets.

ncRNA	Name	Dysregulation	Possible signaling pathways	Pathogenic functions	Reference
circRNA	circ_0084043	upregulated	miR-128-3p/TXNIP, Wnt/β-Catenin; miR-140-3p/TGFA	promote viability inhibition, apoptosis promotion, and inflammatory response of RPEs; promote cell apoptosis of RPEs	(74, 75)
	circ_0000615	upregulated	miR-646/YAP1	promote cell apoptosis, inflammation oxidative stress of RPEs	(76)
	has_circ_0041795	upregulated	miR-646/VEGFC	promote cell apoptosis of RPEs	(9)
	circ-ITCH	downregulated	MMP-2, MMP-9, miR-22	inhibit the neovascularization and inflammation of RPEs	(77)
lncRNA	lncRNA HOTAIR	upregulated	VEGF-A	promote oxidative stress and modulating epigenetic of RPEs	(78)
	LncRNA BDNF-AS	upregulated	BDNF	promote apoptosis of RPEs	(79)
	LncRNA Gas5	downregulated	SERCA2	inhibit ER stress, apoptosis and inflammation of RPEs	(80)
	LncRNA MEG3	downregulated	miR-34a/SIRT1; VEGF	inhibit apoptosis and secretion of inflammation cytokines of RPEs; inhibit development of diabetic retinopathy of RPEs	(81)
miRNA	miR-132	upregulated	occludin, JAK/STA T3	promotes regulate cell viability, mobility and permeability of RPEs	(82)
	miR-219−5p	upregulated	LRH-1/Wnt/β-Catenin	promote apoptosis of RPEs	(83)
	miR-203a-3p	upregulated	SOCS3	promote apoptosis of RPEs	(84)
	miR-423-5p	upregulated	NFE2/miR-423-5p/TFF1	promote apoptosis of RPEs	(84)
	miR−217	upregulated	SirT1	promote apoptosis of RPEs	(85)
	miR-125b	downregulated	HK2	inhibit apoptosis of RPEs	(86)
	miR-200-3p	downregulated	TGF-β2/Smad	inhibit cell proliferation and reduces apoptosis of RPEs	(87)
	miR-411	downregulated	ROBO4	inhibit apoptosis of RPEs	(88)
	miR-146a-5p	downregulated	ROBO4	inhibit decreased cell viability, enhanced permeability, and increased cell migration of RPEs	(89)
	miR-455-5p	downregulated	SOCS3	inhibit oxidative stress of RPEs	(84)

### miRNA-Mediated Mechanism

Currently, miRNAs have been reported to play a key role in regulating RPE apoptosis, proliferation, and migration. Abnormally expressed miRNAs promote pathological changes in the retinal pigment epithelium by activating different signaling pathways. Some miRNAs play a role in promoting RPE damage. For example, miR-132 is highly expressed in the HG environment and targets occludin to activate the JAK/STAT3 pathway, which increases the mobility and permeability of RPE and decreases its survival rate (82). Suppressors of cytokine signaling (SOCS3) are involved in the endoplasmic reticulum stress pathway of tumor cells (90). Under hypoxic conditions induced by high glucose, Mi-203a-3p can perform targeted inhibition of the expression of SOCS3 and promote RPE apoptosis (91). As a target of miR-218, the transcription factor Runx2 is inhibited, promoting RPE apoptosis in the HG state (92). The transcription factor NFE2 can directly upregulate the activity of miR-423-5p, regulate the expression of TFF1 in RPE cells in the HG environment, and activate the NF-κB pathway, aggravating RPE damage (85). This result suggests that the NFE2/miR-423-5p/TFF1 axis plays a role in HG-induced apoptosis in RPE. Similarly, miR-217 can downregulate the expression of SIRT1, aggravate the inflammatory response of RPE and activate the NF-κB signaling pathway (93). Zhao et al. found that inhibiting the expression of miR-219-5p can significantly increase the activity of RPE. The damaging effect of miR-219-5p on RPE may be caused by the activation of Wnt/β-catenin by LRH-1 (83), while LRH-1 plays a key role in the regulation of apoptosis (94).

In addition to the abovementioned miRNAs that promote RPE damage, the expression of some miRNAs will also protect RPE in the DR state. HG induction downregulates miR-200-3p in RPE, and further research proves that miR-200-3p can reduce the inflammatory effect by inhibiting the TGF-β2/Smad signaling pathway, which provides a new target for DR treatment in the clinic (87). miR-411 plays a protective role in HG or RPE in a hypoxic state. It can downregulate the expression of ROBO4 and reduce the permeability of RPE, thereby improving the survival rate of RPE (88). Another study showed that ROBO4 in RPE can be inhibited by miR-125b-5p in the HG state, while ROBO4 in RPE can be inhibited by miR-146a-5p in the hypoxic state (89). It is suggested that different stresses in the HG state will lead to the activation of different signaling pathways in RPE, and miR-411, miR-125b-5p, and miR-146a-5p may all target ROBO4 to protect RPE. A previous article showed that miR-203a-3p targeting SOCS3 damages PRE, but another study found that SOCS3 is also the target of miR-455-5p, and overexpression of miR-455-5p can downregulate SOCS3 to protect RPE (84). Under HG conditions, the RPE glycolytic pathway is in a disordered state, and miR-125b can target hexokinase 2 (HK2), reducing the glycolytic activity of RPE to delay the progression of DR (86).

### CircRNA-Mediated Mechanism

CircRNAs play an important role in the process of RPE lesions by virtue of their biological properties. CircRNAs can regulate the function and state of cells through a variety of biological functions, such as interacting with proteins or serving as translation templates. However, current evidence has shown that amid the regulation of RPE pathological changes, circRNAs are mainly used as miRNA sponges to regulate the expression level of downstream proteins, thereby utilizing the regulatory network in the process. circ-ITCH was found to be circRNA that was abnormally downregulated in RPE in DR rats (77). circ-ITCH can reduce the expression of miR-22 through sponging miR-22, inhibiting important regulators of angiogenesis, such as matrix metalloproteinases-2 (MMP-2) and MMP-9, and the expression of tumor necrosis factor α (TNF-α) together with other inflammatory factors, alleviating the pathological trend of DR. Qiang et al. (76) discovered a stable circRNA with increased expression under the induction of HG—circ_0000615 that has a miR-646 binding site, which can downregulate the expression level of miR-646 in a targeted manner. After knocking out the circ_0000615 gene, Bcl-2 increased, the activity of Bax and C-caspase3 decreased, the viability of HG-induced RPE cells was restored, the inflammatory response was weakened, and the expression of proinflammatory cytokines decreased (TNF-α, IL-1β, and IL-6). YAP1 is a key effector of the Hippo pathway, and it plays an important role in regulating cell survival (95). Studies have found that YAP1 is a downstream targeted regulatory protein of miR-646. Upregulation of miR-646 can antagonize the inhibition of YAP1 expression after overexpression of circ_0000615, promoting the repair of endothelial cells. Another study on circRNA (9) found that miR-646 has different signal transduction pathways in HG-treated RPE. In this study, miR-646 could be sponged by hsa_circ_0041795 to downregulate the expression, and miR-646 could interact with VEGFC to downregulate the expression of VEGFC. In HG-induced RPE, the overexpression of hsa_circ_0041795 increased VEGFC by downregulating miR-646, which ultimately promoted RPE apoptosis, accelerating the progression of DR. Different circRNAs can regulate the pathological process of RPE through common miRNA, and the same circRNA can also play a role in DR through different miRNAs. Li et al. found (74) that the activity of circRNA_0084043 was significantly upregulated under HG induction and promoted ARPE-19 cell proliferation, HG-induced apoptosis, oxidative stress and inflammatory reactions. Further experiments proved that circRNA_0084043 can target miR-140-3p, which indirectly regulates the expression level of transforming growth factor-A (TGF-A). In the study of Zhang et al. (75), after RPE was treated with HG, the activity of circ_0084043 was upregulated, and the expression of miR-128-3p was inhibited by sponging. TXNIP proved to be a downstream regulator of miR-128-3p. In addition, circRNA_0084043 regulates the Wnt/β-catenin signaling pathway through the miR-128-3p/TXNIP axis. The Wnt/b-catenin signaling pathway is related to the promotion of inflammation and vascular exudation and is involved in the progression of DR (96).

### LncRNA-Mediated Mechanism

Many lncRNAs have been shown to aggravate RPE damage in the HG environment, suggesting that they play an important role in the development of DR. VEGF is a key factor in the formation of new blood vessels during the development of DR, plays an important role in diabetic macular edema and is currently one of the targets of DR treatment (97). Some lncRNAs in RPE can act on VEGF in different ways to regulate the progression of DR. Research (78) discovered that under HG conditions, the expression of lncRNA HOTAIR in the cytoplasm and nucleus of RPE increased. After inhibiting its expression, the expression of a variety of transcripts related to angiogenesis (VEGF-A, ET-1, ANGPTL4, PGF, HIF-1α) and epigenetic regulation (EZH2, SUZ12, DNMT1, DNMT3A, DNMT3B, CTCF, P300) was downregulated, which improved cell survival. In addition, HOTAIR can dynamically regulate the levels of RNA polymerase II and acetylation factor (P300) in the distal and proximal promoter regions of its downstream target VEGF-A by interacting with histone-modifying enzymes, which promotes the epigenetic activation of VEGF-A, leading to the overexpression of VEGF-A and the formation of new blood vessels. Moreover, HOTAIR is also related to vascular permeability in DR, suggesting that lncRNAs may function in the nonproliferative stage. Another study showed that there was a decrease in the expression level of lncRNA MEG3 in the serum of DR patients, and the serum VEGF level was significantly higher than the normal level. Cell experiments have found that the overexpression of MEG3 has a negative regulatory effect on the expression level of VEGF, but the specific form of VEGF regulation has not been clearly studied (81). SERCA is a key molecule for the cytoplasm and endoplasmic reticulum to carry out Ca^2+^ transfer and maintain Ca^2+^ homeostasis (98), and it can regulate Ca^2+^-related signaling pathways. The lncRNA Gas5/SERCA axis was proven to regulate endoplasmic reticulum stress, the inflammatory response, and apoptosis of RPE cells treated with HG, and both Gas5 and SERCA were expressed at low levels in RPE cells treated with HG (80), suggesting that these signaling pathways may act as a protective factor for RPE to inhibit the progression of DR. Brain-derived neurotrophic factor (BDNF) is a type of nerve growth factor that is abundantly expressed in the brain and peripheral system and plays a role in promoting DR (79, 99). LncRNA BDNF-AS is highly expressed in HG-induced RPE and acts on BDNF mRNA to inhibit BDNF expression and promote cell apoptosis (79). In summary, these lncRNAs that play a role in RPE lesions induced by HG may have potential therapeutic effects. Reversing its expression level may delay the damage process of RPE.

## miRNAs and lncRNAs Affect the Development of DR Through Retinal Nerve Cells

**Figure 4:**
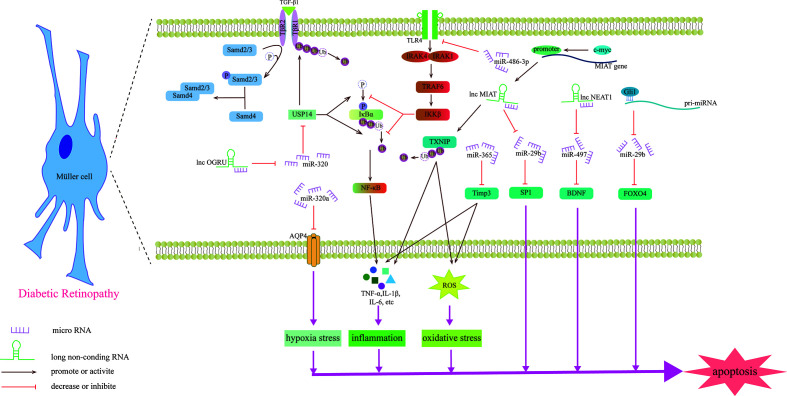
Schematic diagram of the mechanism by which miRNAs and lncRNAs affect Müller cells and participate in the development of DR.

The retinal tissue is composed of blood vessel tissue, neurons, and supporting glial cells. These cells interact to form a structure called the neurovascular unit (NVU) (100). DR has two interrelated components: diabetic retinal neurodegeneration (DRN) and diabetic retinal vascular disease (DRV) (101). Nerve cell apoptosis of ganglion, amacrine and Müller cells and activation of microglia occur in DRN (102). Diabetic patients with early DR, or even before the occurrence of DR, have neurodegeneration such as thinning of the ganglion cell layer (GCL) and retinal nerve fiber layer (RNFL) (103, 104). Previous studies have shown that miRNAs, lncRNAs, and circRNAs can affect the progression of neuropathy in the process of DR (**Figure 4**). We summarized these related research results to provide new ideas for the study and treatment of DR from the perspective of neuropathy.

Previous studies have shown that miRNAs may be involved in the degeneration of Müller cells caused by DR. For example, upregulating the expression of miR-486-3P can protect Müller cells from oxidative stress, inflammation and apoptosis in the HG state and inhibit the TLR4/NF-κB axis (105). Aquaporin-4 (AQP4) can promote the diffusion of water in the cell membrane, and miR-320a can promote the internalization of AQP4, alleviating the edema of Müller cells under hypoxic stress, and may be a potential therapeutic target (106). miR-29a/bs, which are affected by Gli1 and downregulated in the HG state, all target FOXO4 and bind to FOXO4 mRNA to negatively regulate its expression, aggravating the damage to retinal Müller cells (107). The gliosis of Müller cells is positively correlated with the expression of miR-365. In the HG state, miR-365 can downregulate the expression of TIMP3 to promote retinal oxidative stress and gliosis and aggravate DR disease (108). Zhang et al. explored the role of miRNA in retinal ganglion cells and found that miR-495 is involved in ganglion cell apoptosis in the HG state. From a mechanistic point of view, it may be that miR-495 affects the transmission of PTEN/Akt signaling by targeting Notch1, aggravating the damage to ganglion cells (109). S100A12 is a member of the calcium binding protein family. The level of plasma S100A12 relates to the presence of DR, which can activate the inflammatory response of immune cells (110). S100A12 can inhibit the expression of miR-30a, and miR-30a activates retinal microglia in an NLRP3-dependent manner and promotes the progression of DR (111).

Recently, the role of lncRNAs in Müller cells was studied (**Table 3**). OGRU is a type of lncRNA that is highly expressed in Müller cells in the DR state and aggravates the oxidative stress of Müller cells. Exploration of this mechanism has found that OGRU can regulate the expression of USP14 by downregulating miR-320. USP14 directly prevents the ubiquitination and degradation of transforming growth factor-β1 receptors (113). c-myc is the factor that regulates inflammatory mediators (115). In Müller cells, the promoter of lncRNA MIAT can be activated by c-myc. The overexpression of TXNIP caused by upregulated MIAT further activates inflammatory factors (IL-1β, TNF-α and IL-6), aggravating Müller cell damage induced by HG (112). However, another study found that MIAT has a highly homologous target sequence with miR-29b, which can negatively regulate the level of miR-29b and regulate Müller cell apoptosis in DR through the miR-29b/Sp1 axis (116). In addition to the damaging effect, lncRNAs also play a protective role in the nerve cells of DR. For example, upregulating the expression of NEAT1 in Müller cells in the HG state can downregulate miR-497 and increase the expression of BDNF, hindering the damaging effect of HG on Müller cells (114). Some lncRNAs can directly change the morphology of photoreceptors, such as MALAT1, which is upregulated through DR induction, which makes cones appear sparsely arranged and assumes the form of relatively short outer segments. This specific signaling pathway, however, has not been explored (117).

**Table 3:** The role of miRNA ln and lncRNA in DR and their mechanisms through müller cell and targets.

ncRNA	Name	Dysregulation	Possible signaling pathways	Pathogenic functions in Müller cells	Reference
lncRNA	MIAT	upregulated	TXNIP, miR-29b/Sp1	promote inflammation and apoptosis	(112)
	OGRU	upregulated	miR-320/USP14	promote oxidative stress and inflammation	(113)
	NEAT1	downregulated	miR-497/BDNF	inhibit apoptosis	(114)
miRNA	miR-486-3p	downregulated	TLR4/NF-κB	inhibit oxidative stress, inflammation, apoptosis and angiogenesis	(105)
	miR-320a	downregulated	AQP4	inhibit hypoxia injury	(106)
	miR-29a/b	downregulated	FOXO4	decreased the glutamate level	(107)
	miR-365	upregulated	Timp3	promote oxidative stress	(108)

The abnormal expression of circRNA has been shown to be an intermediate phase in a variety of signaling pathways involved in the process of diabetic peripheral neuropathy, especially peripheral nerve pain (118). For example, knocking out circHIPK3 can restore the expression of miR-124, inhibit neuroinflammation and alleviate neuralgia in diabetic rats (119). However, the role of circRNA in nerve cell damage caused by DR has not been verified, and it may be a new research direction.

## Conclusion

DR is a complication of diabetes caused by multiple risk factors, and its pathogenesis is complex. A thorough understanding of the molecular mechanism of DR will help determine new and effective diagnostic and therapeutic targets. In recent years, researchers have discovered that miRNAs, circRNAs, and lncRNAs play an important role in many fields, such as tumors, chronic diseases and related complications. This article reviews some RNAs and their molecular mechanisms that play an important role in the DR progression process of retinal microvascular endothelial cells, retinal pigment epithelial cells and retinal nerve cells. The vast majority of circRNAs and lncRNAs are used as upstream regulators of miRNAs to downregulate the expression of miRNAs, which in turn affects the pathological process of different cells. VEGF is the intersection of many RNAs in DR-related signaling pathways and has an important influence on the DR process. Although a variety of miRNAs, circRNAs, and lncRNAs have been found to be involved in DR disease, such as oxidative stress and endothelial dysfunction, the mechanism of action of many ncRNAs is still unclear, and the same ncRNA may play different roles in different models. Therefore, it is necessary to further study their mode of action in the etiology and pathology of the disease to clarify their role in the pathogenesis of DR. However, a standard for estimating ncRNA activity has not been established, and which specific ncRNA plays a dominant role in regulation needs to be further studied. The ultimate goal of basic research is to solve the problems encountered in clinical work. A deep understanding of these three ncRNAs will help develop new strategies to effectively treat this disease and reduce the chance of blindness due to the progression of retinopathy. The goal of molecular therapy targeting retinal pigment epithelial cells and nerve cells is to inhibit their apoptosis, and for microvascular endothelial cells, the proportion of their apoptosis and regeneration must be controlled. Excessive apoptosis and regeneration will aggravate the progression of DR.

There are some shortcomings in the current research on these three types of RNA. First, DR is a chronic disease. In the process of HG-induced cell modeling, it is impossible to simulate the actual situation of the retinal microcirculation of diabetic patients. Optimizing existing experimental methods to simulate HG-induced lesions as much as possible may be more helpful for clinical treatment. Studies on the early stage of DR are not as comprehensive as those on PDR. Second, there are few studies on circRNAs in DR, especially on the mechanism in retinal neuropathy, and the functions of circRNAs, other than miRNA sponges, have not been explored in DR. Finally, most of the exploration targeting RNAs in retinal neuropathy is focused on Müller cells, while there are few studies on rods, cones, bipolar cells, and ganglion cells. Given that neuropathy exists in the early stages of DR, it may emerge as a relatively influential therapeutic target. In summary, the deficiencies of current research may offer directions for future exploration and contribute to the improvement of the understanding of the pathogenesis of DR.

## Author Contributions

Conceptualization and wrote the original draft of the manuscript, XC and GZ. Methodology, ZC. Software, YW and RL. Validation, XT and CM. Conceptualization and Funding acquisition, SF. All authors contributed to the article and approved the submitted version.

## Funding

The Project of Local Science and Technology Development guided by the Central Government (innovative platform for improving the Prevention and Treatment of Multiple Diseases in Gansu), the Construction Plan of Gansu Province Clinical Research Center for Endocrine Disease (20JR10FA667), the Project of Gansu Natural Science Foundation (20JR10RA681), Lanzhou Science and Technology Development guiding Plan Project (2019-ZD-38), College Students’ Innovation, Entrepreneurship and Excellence Program of Lanzhou University in 2020 (20200060103), and College Students’ Innovation and Entrepreneurship in Lanzhou University in 2021 (20210060155).

## Conflict of Interest

The authors declare that the research was conducted in the absence of any commercial or financial relationships that could be construed as a potential conflict of interest.

## Publisher’s Note

All claims expressed in this article are solely those of the authors and do not necessarily represent those of their affiliated organizations, or those of the publisher, the editors and the reviewers. Any product that may be evaluated in this article, or claim that may be made by its manufacturer, is not guaranteed or endorsed by the publisher.
